# Polyarticular extension of pigmented villonodular synovitis to contiguous joints via pigmented villonodular tenosynovitis

**DOI:** 10.1259/bjrcr.20150404

**Published:** 2016-05-25

**Authors:** David McKean, Eeke Thomee, Joseph Papanikitas, Lennard Y W Lee, Philip Yoong, Sarah Yanny, James L Teh

**Affiliations:** ^1^ Department of Radiology, Stoke Mandeville Hospital, Buckinghamshire Healthcare NHS Trust, Aylesbury, UK; ^2^ Department of Radiology, Nuffield Orthopaedic Centre, Oxford University Hospitals NHS Trust, Oxford, UK; ^3^ Wellcome Trust Centre for Human Genetics, Oxford, UK; ^4^ Department of Radiology, Royal Berkshire Hospital, Royal Berkshire NHS Foundation Trust, Reading, UK

## Abstract

Pigmented villonodular synovitis is an uncommon benign neoplastic process that affects synovial-lined joints, bursae and tendon sheaths. We describe polyarticular extension of pigmented villonodular synovitis across joints secondary to pigmented villonodular tenosynovitis. Given that treatment is required to prevent progressive destruction of the involved joint, tendon or bursa, radiologists must be vigilant for diffuse polyarticular or extrasynovial involvement to optimize patient care and initiate appropriate therapy.

## Summary

Pigmented villonodular synovitis (PVNS) is a rare but potentially aggressive condition that may involve all synovial membranes, including joints, bursae (pigmented villonodular bursitis) or tendon sheaths [pigmented villonodular tenosynovitis (PVNTS) or giant cell tumour of the tendon sheath (GCTTS)].

It was first described by Chassaignac^1^ in 1852 as a nodular lesion of the synovial membrane that affects the flexor tendons of the fingers. Jaffe et al^2^ described it as a group of synovial, tenosynovial and bursal lesions. It was then sub-classified into localized and diffuse forms of synovial involvement by Granowitz and colleagues.^3,4^

In most cases, the disease is monoarticular and most commonly involves the knee joint, followed by the hip and ankle joints in order of frequency.^5^ However, PVNS has the potential to extensively invade the local structures such as muscles, tendons, bones and skin.^6^

Polyarticular or multifocal involvement is uncommon.^7–9^ We report a rare case of polyarticular PVNS with involvement of the distal contiguous joints secondary to pigmented villonodular tenosynovial invasion.

## Case report

A 41-year-old female presented to our department with long-standing pain, with development of palpable soft tissue nodules over the volar and dorsal aspect of her right wrist and progressively worsening flexion contractures affecting the middle and ring finger metacarpophalangeal joints (MCPJs) of the right hand. Physical examination confirmed the presence of soft tissue nodules and flexion contractures, with markedly reduced movement of the middle and ring finger MCPJs.

## Investigations

**Figure 1.**
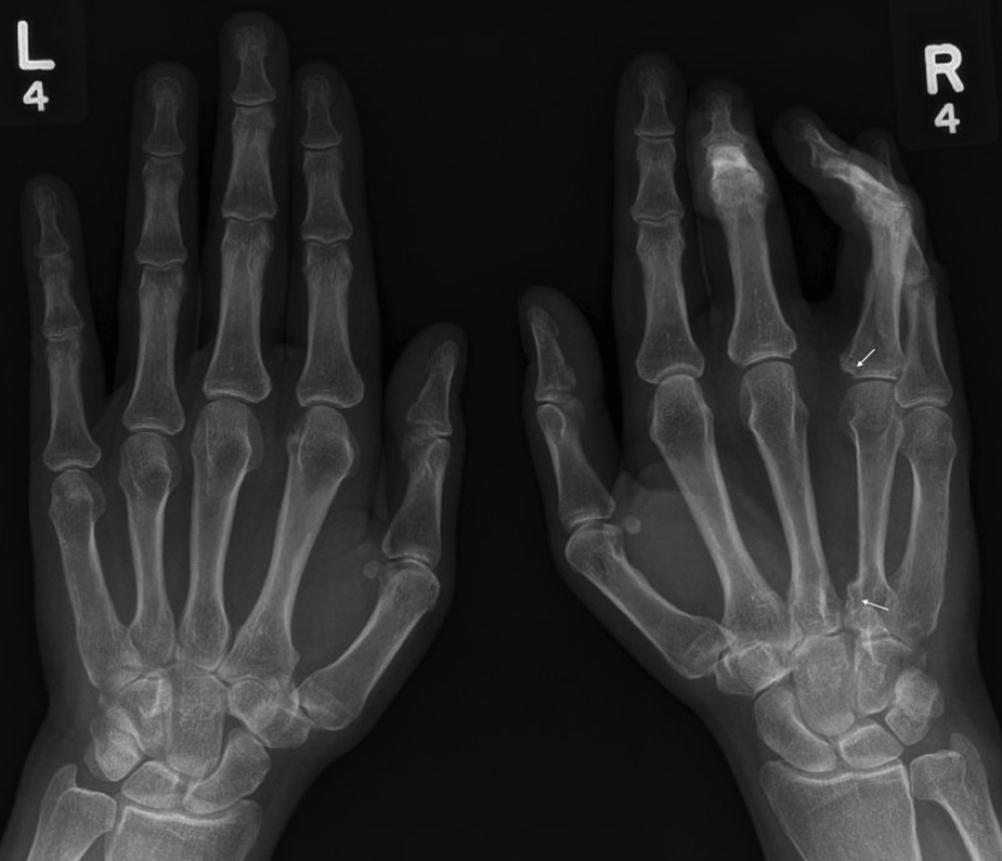
Flexion deformity of the right middle and ring fingers with erosions of the right index and ring finger carpometacarpal joints and metacarpophalangeal joints) evident (arrows).

A plain film demonstrated flexion deformity of the middle and ring fingers with erosions at the proximal interphalangeal joint of the ring finger, early cortical irregularity of the base of the index metacarpal and ring finger proximal phalanx (Figure 1).

**Figure 2.**
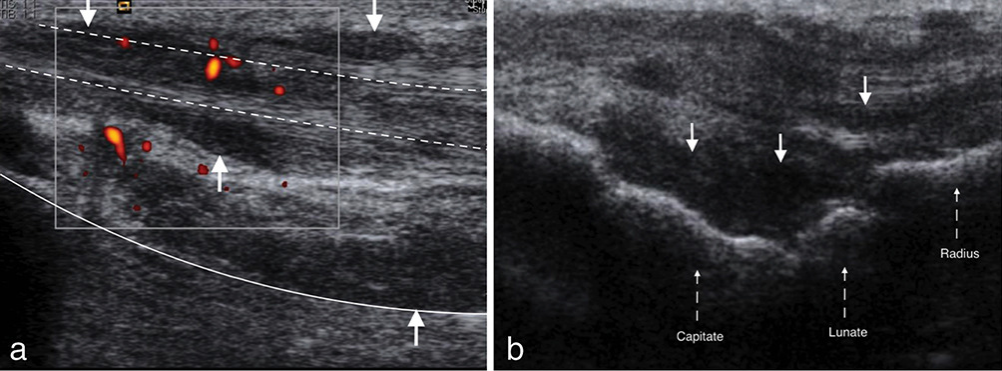
(a) Long-axis power Doppler ultrasound image demonstrating hypoechoic nodules (arrows) and tenosynovitis extending along the flexor tendon sheaths (dashed lines) and overlying the radius (solid line). (b) Long-axis ultrasound demonstrating synovial hypertrophy and hypoechoic nodules (arrows) over the dorsal carpus (dashed arrows).

Ultrasound confirmed the presence of flexion deformities with the palpable nodules corresponding to the flexor tendon sheaths. There was marked tendinosis with associated tenosynovitis affecting the middle and ring finger flexor tendons (Figure 2a). There was particular thickening of the tendon sheath at the level of the A1 pulleys and generalized thickening of the flexor tendons in the palm and the wrist, with compression of the median nerve in the carpal tunnel.

On dynamic imaging, there was poor excursion of the flexor digitorum superficialis tendons of the middle and ring fingers and, to a lesser extent, the flexor digitorum profundus tendons. There was moderate associated vascularity on power Doppler interrogation.

On scanning the dorsum of the wrist, there was again evidence of extensor tenosynovitis with nodular tenosynovial thickening and echogenic material in the tendon sheath. There was synovial hypertrophy at the carpus (Figure 2b) and MCPJs with minor increase in vascularity. No evidence of synovitis or tenosynovitis was seen in the left wrist.

**Figure 3.**
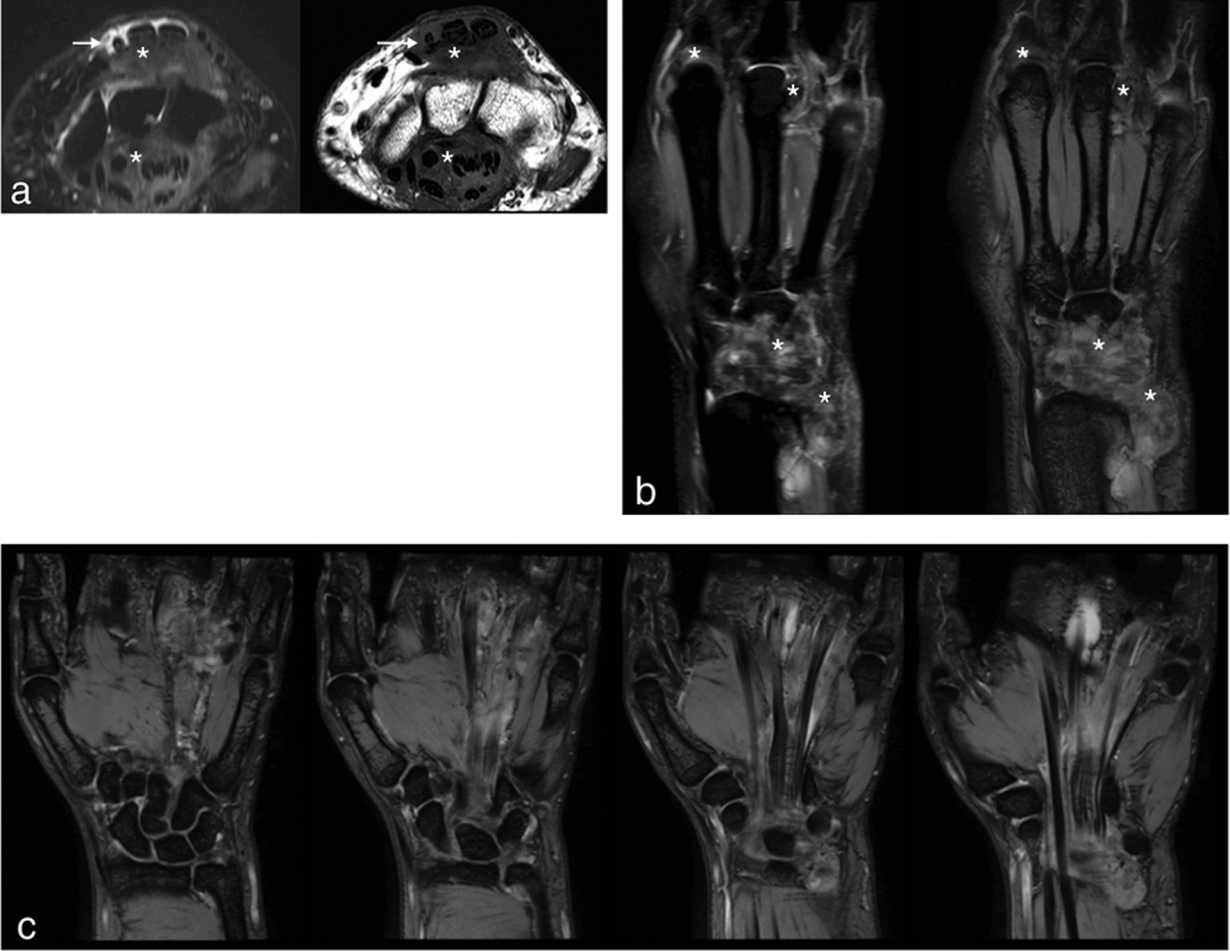
(a) Short tau inversion-recovery and *T*_1_ weighted axial images demonstrating extensive peritendinous soft tissue thickening consistent with florid tenosynovitis affecting the extensor and flexor compartments with mild fluid distension (arrows) and peritendinous low signal (asterisks). (b) Coronal proton density spectral attenuated inversion-recovery and gradient echo sequences demonstrating low signal synovitis with areas of blooming artefact (asterisks) surrounding the wrist, carpus, ring and middle finger MCPJs. (c) Serial coronal gradient echo images demonstrating siderotic lobulated synovial proliferation at the level of the wrist and carpus invading into and tracking distally along the flexor tendons to involve the index, middle and ring finger MCPJs. There are multiple intra-articular and peritendinous foci of low signal with foci of susceptibility artefact consistent with haemosiderin deposition. MCPJs, metacarpophalangeal joints.

An MRI was arranged to further characterize these findings. This revealed florid tenosynovitis affecting the extensor compartments, with intermediate/low signal seen on short tau inversion-recovery and *T*_1_ weighted images with areas of blooming artefact in addition to minor fluid distension (Figure 3a). There was also involvement of the flexor tendons at the level of the carpus, again with low signal surrounding the tendons (Figure 3a). Tenosynovial thickening of the flexor compartment resulted in flattening and compression of the median nerve at the level of the carpal tunnel.

The flexor tendons of the middle and ring fingers were also involved at the level of the MCPJs. Hypointense lobulated synovitis was seen at the wrist and carpus and also at the index, middle and ring finger MCPJs and thumb interphalangeal joint (Figure 3b,c).

Ultrasound-guided biopsy was performed and histological examination confirmed diffuse siderotic tenosynovitis along the tendons involving the MCPJs. This pathology–imaging correlation confirmed the diagnosis of extensive, invasive diffuse PVNS of the adjacent tendon sheaths (PVNTS). Our patient proceeded to have debulking surgery with follow-up for consideration of adjuvant treatment with external beam radiation therapy in the future.

## Discussion

Polyarticular involvement is an unusual presentation of PVNS. While the condition may be locally destructive, to the best of our knowledge, this is the first reported case of contiguous joint involvement *via* tenosynovial extension.

The varying forms of this condition may be divided into localized and diffuse types. The localized subtype is the most common and is usually extra-articular^10^ in contrast to the diffuse form, which is normally intra-articular. The diffuse form, which involves all of the contiguous synovium, is typically mono-articular, with large synovial surfaces most frequently affected such as the knee or hip. Localized intra-articular, or nodular, PVNS is less common and typically occurs in smaller joints. Localized disease, including both extra-articular and intra-articular, represents 77% of cases (with the tenosynovial form being the most common). Localized intra-articular involvement represents 6% of all cases.^11^ Localized extra-articular form of the disease accounts for 1.6–3.9% of all benign soft tissue masses, and is exceeded in frequency only by ganglia.^11,12^

Patients most commonly present in the third to fifth decades of life (mean age late 30s). The diffuse intra-articular form of the disease occurs with equal frequency in both sexes, whereas there is a mild female predilection in the localized extra-articular form (1.5–2.1 : 1).^11^

The current World Health Organization nomenclature describes PVNTS, or pigmented villonodular bursitis, as GCTTS and the diffuse intra-articular form of PVNS as diffuse-type giant cell tumour. These conditions are rare with an incidence estimated to be 9.2 and 1.8 cases per million for extra-articular and intra-articular forms of the disease, respectively.^13^

The pathogenesis of PVNS is likely multifactorial, although cytogenetic analysis of the lesions have demonstrated that the condition may be associated with specific chromosomal aberrations. Translocations are often noted and result in a fusion gene with a strong collagen promoter (*COL6A3*) driving the colony-stimulating factor-1 (*CSF-1*) gene. This results in excessive production of the pro-inflammatory CSF1 protein in collagen and subsequent macrophage and monocyte invasion.^14–16^ However, these changes, while unique, do not fully account for all cases and genome-wide microarray analysis has demonstrated that there is up-regulation of a number of genes involved in apoptosis, matrix degradation and inflammation.^17^

Macroscopically, the synovium is diffusely thickened in PVNS. This synovial hypertrophy consists of irregular papillary or villous projections and larger nodular or villonodular protrusions.

At microscopy, localized extra-articular disease typically reveals a multinodular process embedded in a dense, partially collagenous pseudocapsule.^10^ In contrast, diffuse intra-articular PVNS has an infiltrative sheet-like growth pattern tracking along the synovium with the potential for local invasion. These masses are typically dark brown and heterogeneous in colour with areas of yellow discolouration (xanthoma cells). The classic cytoarchitecture consists of subsynovial nodular proliferation of large round, polyhedral or spindle cells with prominent cytoplasm and pale nuclei. The multinucleated giant cells contain a variable number of nuclei, from 3 or 4 to 50.^18^ The multinucleated giant cells are less conspicuous and not as uniformly dispersed in diffuse intra-articular PVNS and may be absent in up to 20% of cases. Overall, there is a diffuse infiltrative growth pattern with the potential for local invasion.^10^ The histological appearance may mimic aggressive neoplasms such as synovial sarcoma, rhabdomyosarcoma or epithelioid sarcoma. Malignant transformation of PVNS is rare, and controversy exists regarding the histological criteria for its diagnosis.^19^ Imaging alone is not sufficient for diagnosis, and pathologic imaging correlation is necessary for the exact diagnosis and for differentiating between the benign and the malignant forms of the disease.^11,19–21^

The typical features on plain film are relatively non-specific, with appearances mainly being those of a joint effusion. Bone density and joint space are preserved until late stages. Marginal erosions may be present as in our case (Figure 1) but it is not possible to distinguish PVNS from other primary synovial processes such as synovial chondromatosis.^20^

On ultrasound, intra-articular PVNS has non-specific imaging features and may appear as a heterogeneous echogenic mass or markedly thickened hypoechoic synovium that may have nodular and villous projections with internal vascularity on Doppler interrogation.^10,20,21^ PVNTS or GCTTS appears as a hypoechoic solid mass with well-defined margins and internal vascularity that is intimately related to the associated involved tendon.^20,21^ As the mass does not arise from the tendon sheath, GCTTS do not move with the tendon during dynamic imaging.^10,20,21^

On CT scan, joint effusions are commonly seen. The hypertrophic synovium appears as a soft tissue mass that may appear slightly hyperdense compared with adjacent muscle secondary to haemosiderin content. Calcification is very rare in the synovial mass in contrast to synovial sarcoma, where this is common.^21^ Articular erosions may also be evident.

MRI typically demonstrates a mass-like synovial proliferation with lobulated margins.^10,22^ This may be extensive in the diffuse form or limited to a well-defined single nodule in the localized form. The signal characteristics include low-to-intermediate signal on *T*_1_ weighted images, variable enhancement following gadolinium,^23^ low-to-intermediate signal on *T*_2_ weighted images with some areas of high signal owing to joint fluid or an inflamed synovium. Gradient echo sequences demonstrate low signal and may reveal blooming artefact owing to haemosiderin. The differential diagnosis would include siderotic synovitis,^24,25^ and predisposing conditions such as bleeding disorders or synovial haemangioma should be excluded.

PVNS may be locally invasive and involvement of the adjacent bone can result in pathological fracture.^26–28^ Contiguous spread involving the joints of the carpus or tarsal bones may also be seen. While our description of poly-articular PVNS with invasion of the adjacent tendons and spread to distal contiguous joints has not previously been reported, the potential for local invasion and extension along tendon sheaths as with GCTTS is well documented. Such aggressive patterns of spread may be seen in other malignant conditions, such as epithelioid sarcomas, which are extremely malignant and tend to spread along the tendon sheath, fascial planes and lymphatics.^29^ Myxoid chondrosarcoma may also be associated with the tendon sheath but is usually seen in the digits and more commonly present in older males.^30^

## Conclusions

In summary, PVNS is a rare synovial disorder. The typical features of this disease on MRI are characteristic and aid in differentiating it from other synovial proliferative processes. The characteristic blooming seen on gradient recalled echo images and the low *T*_1_ and *T*_2_ signal intensity reflect the underlying histological features of haemosiderin deposition within the lesion. There is potential for local invasion and rarely, as in our case, spread to contiguous joints. The identification of such atypical patterns of spread is essential to guide appropriate treatment to prevent progressive destruction of the involved joint, tendon or bursa.

## Learning points

1. PVNS and PVNTS, also known as GCTTS, are rare synovial disorders of uncertain aetiology with characteristic imaging findings.
2. Pathology–imaging correlation is necessary for accuate diagnosis.
3. There is potential for local invasion and extension from the affected joint into the adjacent tendon sheaths.
4. Our case demonstrates that advancement of this giant cell tumour along the tendon sheaths may provide a route for involvement of distant joints.
5. The identification of such atypical patterns of spread is essential to guide appropriate treatment to prevent progressive destruction of the involved joint, tendon or bursa.

## Consent

Informed consent was obtained and is held on record.

## References

[1] Chassaignac M Cancer de la gaine des tendons. Gas Hosp Civ Milit 1852; 47: 185–90.

[2] Jaffe HL, Lichtenstein L, Sutro CJ Pigmented villonodular synovitis: bursitis and tenosynovitis. Arch Pathol 1941; 31: 731–65.

[3] Granowitz SP, D'Antonio J, Mankin HL The pathogenesis and long-term end results of pigmented villonodular synovitis. Clin Orthop Relat Res 1976; 114: 335–51. 770040

[4] Flandry F, Hughston JC Pigmented villonodular synovitis. J Bone Joint Surg Am 1987; 69: 942. 3597511

[5] Dorwart RH, Genant HK, Johnston WH, Morris JM Pigmented villonodular synovitis of synovial joints: clinical, pathologic, and radiologic features. AJR Am J Roentgenol 1984; 143: 877–85. 6332499 10.2214/ajr.143.4.877

[6] Ray RA, Morton CC, Lipinski KK, Corson JM, Fletcher JA Cytogenetic evidence of clonality in a case of pigmented villonodular synovitis. Cancer 1991; 67: 121–5. 1985707 10.1002/1097-0142(19910101)67:1<121::aid-cncr2820670122>3.0.co;2-p

[7] Tavangar SM, Ghafouri M Multifocal pigmented villonodular synovitis in a child. Singapore Med J 2005; 46: 193–5. 15800727

[8] Vedantam R, Strecker WB, Schoenecker PL, Salinas-Madrigal L Polyarticular pigmented villonodular synovitis in a child. Clin Orthop Relat Res 1998; 348: 208–11. 9553554

[9] Wagner ML, Spjut HJ, Dutton RV, Glassman AL, Askew JB Polyarticular pigmented villonodular synovitis. AJR Am J Roentgenol 1981; 136: 821–3. 6784483 10.2214/ajr.136.4.821

[10] Murphey MD, Rhee JH, Lewis RB, Fanburg-Smith JC, Flemming DJ, Walker EA Pigmented villonodular synovitis: radiologic-pathologic correlation. Radiographics 2008; 28: 1493–518. 18794322 10.1148/rg.285085134

[11] Kransdorf MJ, Murphey MD Synovial tumors In: Imaging of soft tissue tumors. Philadelphia, PA: Lippincott Williams & Wilkins; 2006 381–436.

[12] Hughes TH, Sartoris DJ, Schweitzer ME, Resnick DL Pigmented villonodular synovitis: MRI characteristics. Skeletal Radiol 1995; 24: 7–12. 7709261 10.1007/BF02425937

[13] Myers BW, Masi AT, Feigenbaum SL Pigmented villonodular synovitis and tenosynovitis. Adv Exp Med Biol 1980; 59: 223–38. 7412554

[14] Ohjimi Y, Iwasaki H, Ishiguro M, Kaneko Y, Tashiro H, Emoto G, et al Short arm of chromosome 1 aberration recurrently found in pigmented villonodular synovitis. Cancer Genet Cytogenet 1996; 90: 80–5. 8780753 10.1016/0165-4608(96)00064-7

[15] Möller E, Mandahl N, Mertens F, Panagopoulos I Molecular identification of COL6A3-CSF1 fusion transcripts in tenosynovial giant cell tumors. Genes Chromosomes Cancer 2008; 47: 21–5. 17918257 10.1002/gcc.20501

[16] Cupp JS, Miller MA, Montgomery KD, Nielsen TO, O'Connell JX, Huntsman D, et al Translocation and expression of CSF1 in pigmented villonodular synovitis, tenosynovial giant cell tumor, rheumatoid arthritis and other reactive synovitides. Am J Surg Pathol 2007; 31: 970–6. 17527089 10.1097/PAS.0b013e31802b86f8

[17] Finis K, Sültmann H, Ruschhaupt M, Buness A, Helmchen B, Kuner R, et al Analysis of pigmented villonodular synovitis with genome-wide complementary DNA microarray and tissue array technology reveals insight into potential novel therapeutic approaches. Arthritis Rheum 2006; 54: 1009–19. 16508983 10.1002/art.21641

[18] Somerhausen NS, Cin P Giant cell tumour of tendon sheath and diffuse-type giant cell tumour In: Fletcher CDM, Krishnan Unni K, Mertens F, eds Pathology and genetics of tumours of soft tissue and bone. Lyon, France: IARC; 2002 110–14.

[19] Layfield LJ, Meloni-Ehrig A, Liu K, Shepard R, Harrelson JM Malignant giant cell tumor of synovium (malignant pigmented villonodular synovitis). Arch Pathol Lab Med 2000; 124: 1636–41. 11079016 10.5858/2000-124-1636-MGCTOS

[20] Murphey MD, Vidal JA, Fanburg-Smith JC, Gajewski DA Imaging of synovial chondromatosis with radiologic-pathologic correlation. Radiographics 2007; 27: 1465–88. 17848703 10.1148/rg.275075116

[21] Murphey MD, Gibson MS, Jennings BT, Crespo-Rodríguez AM, Fanburg-Smith J, Gajewski DA From the archives of the AFIP: imaging of synovial sarcoma with radiologic-pathologic correlation. Radiographics 2006; 26: 1543–65. 16973781 10.1148/rg.265065084

[22] Masih S, Antebi A Imaging of pigmented villonodular synovitis. Semin Musculoskelet Radiol 2003; 7: 205–16. 14593562 10.1055/s-2003-43231

[23] Barile A, Sabatini M, Iannessi F, Di Cesare E, Splendiani A, Calvisi V, et al Pigmented villonodular synovitis (PVNS) of the knee joint: magnetic resonance imaging (MRI) using standard and dynamic paramagnetic contrast media. Report of 52 cases surgically and histologically controlled. Radiol Med 2004; 107: 356–66. 15103287

[24] Narváez JA, Narváez J, Ortega R, De Lama E, Roca Y, Vidal N Hypointense synovial lesions on T2-weighted images: differential diagnosis with pathologic correlation. AJR Am J Roentgenol 2003; 181: 761–9. 12933477 10.2214/ajr.181.3.1810761

[25] O'Connell JX Pathology of the synovium. Am J Clin Pathol 2000; 114: 773–84. 11068553 10.1309/LWW3-5XK0-FKG9-HDRK

[26] Finn MA, McCall TD, Schmidt MH Pigmented villonodular synovitis associated with pathological fracture of the odontoid and atlantoaxial instability. Case report and review of the literature. J Neurosurg Spine 2007; 7: 248–53. 17688068 10.3171/SPI-07/08/248

[27] Zwass A, Greenspan A, Green SM Giant cell tumor of the tendon sheath with a pathologic phalangeal fracture. A rare association. Bull Hosp Jt Dis Orthop Inst 1985; 45: 87–93. 2990623

[28] Lynskey SJ, Pianta MJ MRI and thallium features of pigmented villonodular synovitis and giant cell tumours of tendon sheaths: a retrospective single centre study of imaging and literature review. Br J Radiol 2015; 88: 20150528. 26440548 10.1259/bjr.20150528 PMC4984941

[29] Peimer CA, Smith RJ, Sirota RL, Cohen BE Epithelioid sarcoma of the hand and wrist: patterns of extension. J Hand Surg Am 1977; 2: 275–82. 330614 10.1016/s0363-5023(77)80126-3

[30] Abramovici LC, Steiner GC, Bonar F Myxoid chondrosarcoma of soft tissue and bone: a retrospective study of 11 cases. Hum Pathol 1995; 26: 1215–20. 7590695 10.1016/0046-8177(95)90196-5

